# Specialty Grand Challenges in Supramolecular Chemistry

**DOI:** 10.3389/fchem.2017.00083

**Published:** 2017-10-17

**Authors:** Tony D. James

**Affiliations:** Department of Chemistry, University of Bath, Bath, United Kingdom

**Keywords:** supramolecular chemistry, specialty grand challenge, molecular machines and motors, chemosensors, dynamic combinatorial chemistry, supramolecular polymers

## Introduction

What do we mean when we talk about Supramolecular Chemistry? The easiest way to describe supramolecular chemistry is to consider the concept of “chemistry beyond the molecule”—We can think of supramolecular chemistry as more than the sum of the parts. In other words the way molecules interact and come together generates new and exciting properties that surpass those of the molecule. The very core of Supramolecular Chemistry, is therefore built using intermolecular bonds. These intermolecular bonds and/or forces help generate assemblies or discreet complexes Such intramolecular forces are the driving force for mechanically interlocked molecules (MIMs) and a new paradigm in supramolecular interactions the mechanical bond (Stoddart, 2009).

## The origins of supramolecular chemistry

The roots of Supramolecular Chemistry can be clearly seen in the “lock and key” principle of Emil Fischer, with enzyme-substrate interactions being the forerunner of molecular recognition and host-guest chemistry (Fisher, 1902). Concomitant with these developments the nature of inter-molecular interactions were being uncovered, such as van der Waals forces, electrostatic interactions, and hydrogen bonding.

Using the increased understanding of molecular complementarity and interactions scientists began to understand the nature of biological structures and processes. Take for example the double helix structure of DNA, where the correct structural representation was only possible when it was realized that two strands of DNA were held together by the hydrogen bonding of complementary nucleotides. Perhaps more importantly, hydrogen bonding was essential to the function of DNA since the dynamic nature of the bonding allowed for self-replication.

In the 1960s the serendipitous discovery of crown ethers by Charles J. Pedersen. Quickly led to the exploration of noncovalent bonds by Chemists including Donald J. Cram and Jean-Marie Lehn. This was the birth of Supramolecular Chemistry. Supramolecular Chemistry grew very quickly and by the 1980s the field boasted many shape- and ion-selective receptors, as well as mechanically interlocked molecular architectures.

Due to these great advances in the understanding of “*chemistry beyond the molecule* the area of Supramolecular Chemistry was recognized through the Nobel Prize for Chemistry in 1987 (Cram et al., 1987). Presented to Donald J. Cram, Jean-Marie Lehn, and Charles J. Pedersen for their seminal contributions to Supramolecular Chemistry and in particular, for the development of “host-guest” chemistry (Lehn, 1988, 1990).

Thirty years later the definition proposed by Jean-Marie Lehn in his Nobel Lecture still holds true (Lehn, 1987).

“*Supramolecular chemistry may be defined as “chemistry beyond the molecule,” bearing on the organized entities of higher complexity that result from the association of two or more chemical species held together by intermolecular forces. Its development requires the use of all resources of molecular chemistry combined with the designed manipulation of non-covalent interactions so as to form supramolecular entities, supermolecules possessing features as well delined as those of molecules themselves. One may say that supermolecules are to molecules and the intermolecular bond what molecules are to atoms and the covalent bond*.”

The only addendum is to extend the definition to include dynamic covalent bonds and the mechanical bond.

Today, Supramolecular Chemistry consists of many rapidly expanding fields that interface with biological chemistry, materials science; synthetic chemistry and facilitates the development improved analytical methods. If we consider the vast scope of Supramolecular Chemistry it is impossible to cover all the grand challenges that are possible from this rich field. However, from a personal perspective, I want to introduce four areas that I believe will be pivotal over the next decade.

## Molecular machines and motors

The first grand challenge I want to consider is part of what I will call the second phase of Supramolecular Chemistry and belongs to “molecular machines and motors” which can also be discussed under the umbrella of nanotechnology (Kay et al., 2007; Erbas-Cakmak et al., 2015).

Two very different kinds of molecular machine exist: molecular switches (or shuttles) and molecular motors. The two systems differ in that a switch changes states, such that switching from “on” to “off” and back to “on” returns the system to the original state. However, when a motor does work in a particular direction, for example a full clockwise rotation that returns everything to the same position any work can only be undone by an identical counter-clockwise rotation.

In 2016 this fledgling area of Supramolecular Chemistry was recognized by the Nobel Prize in chemistry awarded to Jean-Pierre Sauvgage, J. Fraser Stoddart and Ben. L. Feringa (Sauvage et al., 2016) The Nobel Prize to the area of molecular machines indicates the importance of this area and will certainly encourage new research in the area to achieve even greater achievements that will eventually result in molecular robots. These molecular robots will be used for a myriad of tasks including the repair of our bodies at a molecular level (Balasubramanian et al., 2011).

Such molecular level repair will become increasingly important as the average age of the world's population continues its upward spiral. “Vastly improved life expectancy, one of the great triumphs of the last century, looks set to be one of great challenges of this one.” In a little over two years (2020), 1.1 million People, 12% of the UK population will be over 65. An aging population causes two main problems: less tax revenue and more spending on social welfare, and health care thus posing an unprecedented challenge to modern society.

## Molecular sensors (chemosensors)

Perhaps the best description of a chemosensor was given by Anthony W. Czarnik, in the ACS Symposium Series publication on “Fluorescent Chemosensors for Ion and Molecule Recognition” (Czarnik, 1993a,b)—“a fluorescent chemosensor is a compound of abiotic origin that complexes to an analyte reversibly with concomitant fluorescence signal. The technological driving force to achieve useful chemosensors will stimulate investigation of new topics in molecule recognition, fluorescent signal transduction, and their intersection.”

Molecular chemosensors are a branch of Supramolecular Chemistry very close to my heart and represents a very important and thriving area (Chan et al., 2012; Banerjee et al., 2013; Yang et al., 2013b; Carter et al., 2014; Zhang et al., 2014; Ashton et al., 2015; Chang et al., 2015a,b; Lee et al., 2015; Wu et al., 2015, 2017; He et al., 2017a,b). The grand challenges for chemosensors will be to make the transition from research tools into practically useful systems. I believe that this change is already happening with selective chemosensors being developed for an ever increasing spectrum of targets, with enhanced selectivity and improved spectral properties. These enhanced chemosensors coupled with improved instrumentation will lead to better and quicker disease diagnosis. Molecular chemosensors will also lead to new and improved therapeutics either through simple monitoring of patient progress or using theranostics where the therapy and diagnostic system is directly coupled (Kim et al., 2009; Barreto et al., 2011; Yang et al., 2013a). Again, the development of improved chemosensors and theranostic systems will lead to better disease treatments as we move to more personalized medicines.

## Dynamic combinatorial chemistry (DCC)

Another particularly contemporary area is dynamic combinatorial chemistry (also known as dynamic covalent chemistry). Here, as researchers we can take advantage of Supramolecular self-assembly to generate new molecules and materials (Rowan et al., 2002; Cougnon and Sanders, 2012; Wilson et al., 2014). These new systems could be better therapeutic drugs that have been built using dynamic combinatorial chemistry for a specific disease. This for example may use the dynamic assembly of appropriate drug molecules within the active site of a biological target (Ramström and Lehn, 2002; Cougnon and Sanders, 2012; Mondal and Hirsch, 2015). Or we could use dynamic covalent chemistry to develop and prepare materials with improved performance (Belowich and Stoddart, 2012) and properties. One particularly important class of materials are covalent-organic frameworks. These new materials are possible thanks to DCC which assembles the units reversibly resulting in beautifully elegant high-symmetry structures (Waller et al., 2015; Hasell and Cooper, 2016; Diercks and Yaghi, 2017).

## Supramolecular polymers

Supramolecular polymers contain monomer units that are linked via highly directional and reversible non-covalent interactions. These reversible non-covalent interactions, ensure that supramolecular polymers exhibit dynamic properties, including the ability to self-heal. Self-healing polymers could have many applications such scratch resistant coatings, since once scratched they could repair themselves (Aida et al., 2012).

The tuneable and reversible nature of supramolecular systems results in materials with properties that span the nano- and macroscopic scales. These properties include: modularity; mechanical tuneability; and dynamic responsiveness. When applied as biomaterials these properties result in materials that can be used for drug delivery, engineered cell microenvironments, regenerative medicine, and immuno-engineering (Webber et al., 2016).

## Overview

The areas described above are ones close to my heart however Supramolecular Chemistry continues to grow in many directions and my vision for the Supramolecular Section is one where we should move quickly to embrace these new developments. While actively looking to advance these new areas of Supramolecular Chemistry as they evolve the section will not lose sight of the bedrock of Supramolecular Chemistry. The bedrock being core and fundamental developments in practical and theoretical experiments and methods. Keeping, these fundamental aspects in mind will ensure that the section grows to be a trusted resource for all Supramolecular Chemists.

“*I can live with doubt and uncertainty and not knowing. I think it is much more interesting to live not knowing than to have answers that might be wrong. If we will only allow that, as we progress, we remain unsure, we will leave opportunities for alternatives. We will not become enthusiastic for the fact, the knowledge, the absolute truth of the day, but remain always uncertain … In order to make progress, one must leave the door to the unknown ajar*”

Richard P. Feynman (1918–1988).

These words resonate as we set out on the path to develop the new Supramolecular Chemistry Section of Frontiers in Chemistry. Our desire is to become the go to place for readers and authors of new and exciting discoveries, while striving to keep the door to the “unknown ajar.” My overarching desire is to progress the area of Supramolecular Chemistry whilst always remaining open “opportunities for alternatives.” Following these simple ideals I am confident that section will quickly become a trusted source of Supramolecular Chemistry Research.

## Author contributions

The author confirms being the sole contributor of this work and approved it for publication.

### Conflict of interest statement

The author declares that the research was conducted in the absence of any commercial or financial relationships that could be construed as a potential conflict of interest.
